# Orienting towards social features in naturalistic scenes is reflexive

**DOI:** 10.1371/journal.pone.0182037

**Published:** 2017-07-25

**Authors:** Lara Rösler, Albert End, Matthias Gamer

**Affiliations:** 1 Department of Psychology, Julius Maximilians University of Würzburg, Würzburg, Germany; 2 Department of Systems Neuroscience, University Medical Center Hamburg-Eppendorf, Hamburg, Germany; University of Muenster, GERMANY

## Abstract

Saliency-based models of visual attention postulate that, when a scene is freely viewed, attention is predominantly allocated to those elements that stand out in terms of their physical properties. However, eye-tracking studies have shown that saliency models fail to predict gaze behavior accurately when social information is included in an image. Notably, gaze pattern analyses revealed that depictions of human beings are heavily prioritized independent of their low-level physical saliency. What remains unknown, however, is whether the prioritization of such social features is a reflexive or a voluntary process. To investigate the early stages of social attention in more detail, participants viewed photographs of naturalistic scenes with and without social features (i.e., human heads or bodies) for 200 ms while their eye movements were being recorded. We observed significantly more first eye movements to regions containing social features than would be expected from a chance level distribution of saccades. Additionally, a generalized linear mixed model analysis revealed that the social content of a region better predicted first saccade direction than its saliency suggesting that social features partially override the impact of low-level physical saliency on gaze patterns. Given the brief image presentation time that precluded visual exploration, our results provide compelling evidence for a reflexive component in social attention. Moreover, the present study emphasizes the importance of considering social influences for a more coherent understanding of human attentional selection.

## Introduction

In order to successfully navigate in our social environment, it is essential for us to be able to correctly identify and interpret social cues. In a heated debate, one does not only need to recognize the emotion displayed on an opponent’s face to prepare an appropriate response, but prior to doing so, one needs to rapidly allocate attention to the respective face. Although such social attention is crucial to every social skill and interaction, little is known about the neurobehavioral mechanisms enabling it. While numerous studies (e.g. [1–7]) have shown that humans display an attentional bias towards faces or other human features, these studies typically employ a highly controlled design consisting of simplified social stimuli (e.g., schematic or isolated real faces). In the past decade, an increasing number of researchers began questioning the assumption that we can generalize findings from such controlled settings to gaze behavior in real social situations [8,9]. After all, the presentation of an isolated face neglects various challenges entailed in real-life social settings as, for instance, the competition between different social features or with other relevant non-social items in the scene. The studies that have examined gaze patterns in naturalistic scenes have yet shown that humans, and in particular human faces, are still prioritized even when there is competition with other salient objects in the scene (e.g. [10–16]). A question that remains unanswered is whether this prioritization of human features is a reflexive response to the relevant social information or a voluntary reaction possibly driven by the motivational goal of social conformance.

This dichotomy between automatic, bottom-up and controlled, top-down attention has shaped psychological research for decades (as reviewed in [17]). Traditionally, bottom-up processing is believed to be automatically driven by salient stimulus characteristics, which pop out of a scene, whereas top-down processing follows higher cognitive and motivational goals, and is considered a neuroanatomically separate component of attention (as reviewed in [18]). To our knowledge, all studies examining social attention in naturalistic scenes so far have used relatively long presentation times (i.e., several seconds), which does not allow for disentangling these two components. Various attention tasks using simplified social stimuli as well as studies examining primate responses to gaze cues suggest that social attention entails a reflexive component [1,19–22]. Accordingly, if an isolated face is presented next to an inanimate object for varying stimulus onset asynchronies (SOAs) as brief as 100 ms, observers will respond faster to a cue if it appears on the side where the face had previously been shown [20]. Similarly, rhesus macaques were reported to reflexively orient their attention according to the gaze direction of the isolated image of another conspecific’s face [19]. While these findings advocate that social attention is indeed reflexive, it remains to be seen whether this rapid prioritization can also be observed with brief presentations of naturalistic scenes in which social features compete with highly salient non-social regions of an image.

Saliency-based models of attention postulate that attention is automatically oriented to those elements of a picture which stand out in terms of their low-level physical properties (e.g. [23,24], for reviews see [25–27]). Computational algorithms, for example taking into account color, intensity and orientation contrast in an image, can create so-called saliency maps which, in turn, can be used to predict gaze behavior. In previous studies in which participants were asked to freely view or memorize an image, these saliency maps were validated (e.g.[23,28]) and were seen to work particularly well for early fixations, suggesting a predominant modelling of bottom-up attentional processes ([29] but see [30]). Indeed, recent research showed that saliency-based models fail to predict gaze when top-down influences are strong (as reviewed by [31]). Importantly, although prior studies have investigated the impact of social features on the prediction accuracy of these saliency maps, again, the stimuli chosen for the investigation were often not truly representative of naturalistic scenes. As faces frequently presented the focus of the research question, the social features were often found in the foreground of the image rendering a true comparison of gaze behavior towards social and nonsocial features difficult (e.g. [32,33]). Moreover, some studies did not report the saliency values of the social features in their analyses which complicates an interpretation of the separate influences of saliency and social information (e.g. [34]). However, overall, current evidence still suggests saliency maps perform worse when social features are present in the visual field (e.g. [11,13,34]). It yet remains to be seen whether saliency maps perform more accurately when image presentation time is too brief for goal-driven attention to occur.

To investigate whether social attention is truly a reflexive process, we conducted an eye-tracking experiment in which participants viewed complex naturalistic images with and without social features for a brief time period (200 ms) which precluded a detailed visual exploration of the scene. The images were chosen such that social features were always restricted to one quadrant of an image. To avoid that the quadrants containing social features were also the ones with highest low-level saliency in the image, images were carefully selected resulting in balanced physical saliency across all quadrants. We analyzed the direction of the first saccade of each trial and examined whether the presence of social features outperformed physical saliency in predicting saccade direction. We found that observers made significantly more first eye movements towards image quadrants containing social features than a distribution of eye movements at chance level would suggest. In addition, the social content of the quadrant contributed significantly more to saccade direction than its saliency. Considering the brief presentation time of the stimuli, these results support the hypothesis that social attention entails a reflexive component.

## Methods

### Participants

Thirty-nine participants were recruited via an online recruiting system hosted by the University of Würzburg between July and September 2016. Inclusion (age between 18 and 60 years, normal or corrected to normal vision with contact lenses) and exclusion criteria (history of psychiatric or neurological illness) were described on the website allowing participants to self-verify whether they were suitable candidates which was subsequently reconfirmed by the experimenter on the day of the experiment.

A prior power analysis revealed that a sample size of 36 participants was necessary to detect medium effects (*d* = 0.5) in paired comparisons (one-tailed) with a power of 0.9. All participants reported normal or corrected to normal vision. Of the 39 participants, 2 participants were excluded from further analyses because filled-in questionnaires revealed a history of psychiatric or neurological illness. One further participant had to be excluded because of missing data. The final sample consisted of 36 participants (20 males, mean age: *M* = 26.64 years, range: 19–42 years, *SD* = 4.76 years). Ethical approval was obtained by the ethics committee of German Psychological Society (DGPs) and performed in compliance with Declaration of Helsinki guidelines. All participants provided written informed consent and received monetary compensation.

### Stimuli

**Fig 1:**
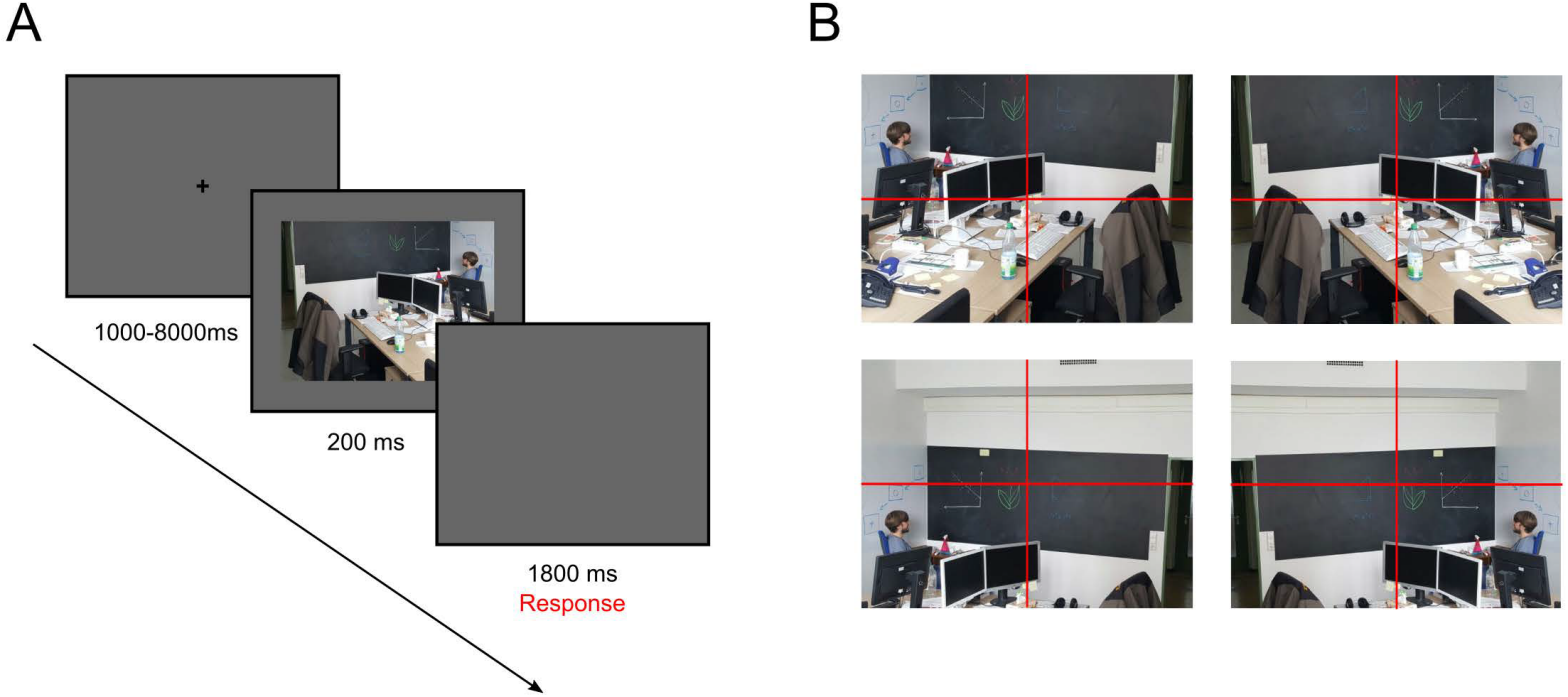
Trial procedure and example stimuli. A) Trial procedure. B) Example image, not included in the original dataset but taken post-hoc, illustrating how stimuli were cropped and mirrored such that social features were being restricted to a different quadrant in each version. The individual depicted in this figure has given written informed consent (as outlined in PLOS consent form) to publish these case details.

The stimulus set consisted of 100 color photographs of complex naturalistic scenes and 5 color target images displaying fractals which we obtained from various image databases (NAPS: [35], Spanky fractal database: http://www.nahee.com/spanky) and the Internet (e.g., Google picture search, flickr). All stimuli were cropped to have the same size of 800 x 600 pixels. The complex naturalistic scenes depicted various indoor and outdoor scenarios. Among these 100 naturalistic scenes, 80 images included parts of one or multiple human beings (social scenes), whereas the remaining 20 images did not contain any human features but instead depicted landscapes or objects (non-social scenes). The social scenes were chosen such that the social features in the scene were largely restricted to one of four quadrants of the image. By mirroring the image and using different cutouts, we were able to create four different versions of the same image displaying the social feature once in each quadrant (see Fig 1B). Written text was removed from the images using the software GIMP (Version 2.8.10, GNU Image Manipulation Program, The GIMP Team) because it would have appeared unusual in the mirrored images. Based on the saliency-algorithm developed by Koch and Ullman [36] and first implemented to gaze behavior in naturalistic scenes by Itti, Koch and Niebur [23,24], we calculated and ranked the relative mean saliency of each quadrant per image. Subsequently, for each participant, one version of each image was pseudo-randomly chosen while ensuring that social elements appeared equally often in one of the four quadrants (20 trials each) and that saliency ranks across all social quadrants were balanced and did not differ systematically from non-social quadrants within each subject (i.e., the average saliency rank of social quadrants amounted to 2.5 at each of the four social feature positions for each subject).

### Apparatus

Participants were seated in a dimly lit room with their head stabilized at 50 cm distance to the computer screen. Stimulus presentation and response collection was controlled by the software Presentation 17.0 (Neurobehavioral Systems, Inc., Berkeley, CA, USA). Each stimulus was displayed centrally on a grey background of a 24” LG 24MB 65PY-B screen (516.5 x 323.1 mm; 1920 x 1200 pixels, 54.63° x 35.81° visual angle, 60 Hz). With this setup, the visual angle of the images amounted to 24.29° x 18.35°. Eye movements were recorded at a sampling rate of 1000 Hz of the right eye of each participant (EyeLink 1000 Plus, SR Research, Ontario, Canada).

### Design and procedure

In order to ensure that attention was paid to stimulus presentation at all times, participants were instructed to press a button upon the appearance of a fractal image. Prior to the experiment, participants underwent a training sequence consisting of twelve practice trials with different stimuli including one fractal image. The actual experiment was performed in two subsequent blocks to avoid fatigue. Each block consisted of 40 social, 10 non-social and 3 fractal images (one was used twice per participant) in pseudorandomized order. The entire experiment thus entailed a total of 106 trials. Because of practical circumstances, one participant performed the experiment in one go and viewed one fractal less (105 trials in total). Each trial started with a fixation cross shown for a random period between 1 and 8 seconds, followed by the stimulus image displayed for 200 ms and a subsequent blank screen shown for 1800 ms during which a response could be made (see Fig 1A).

Following the eye tracking experiment, participants filled in various psychometric tests and questionnaires which will be pooled across several studies and are not part of this manuscript.

### Data processing

Saccades and fixations were detected on a trial-by-trial basis. Accordingly, saccades were defined as eye movements surpassing a velocity threshold of 30°/s or an acceleration threshold of 8,000°/s^2^. These eye movement data were then processed in R (Version 3.3.2, Core Team, 2016). To facilitate drift correction and to ensure baseline stability in all trials entering the analysis, we considered the last 300 ms before stimulus onset (where a central fixation cross was shown) as baseline. For each participant, we examined baseline stability by conducting an iterative outlier removal procedure separately for x- and y-baseline coordinates (for a similar procedure see [13]). Specifically, the smallest and the largest values were temporarily removed from the distribution. If any of these extreme values was more than three standard deviations away from the mean baseline position of the remaining data, it was permanently excluded from the analysis and this procedure was repeated until no more exclusion had to be performed. Saccade x and y coordinates were then corrected for gaze drift by subtracting the baseline from the actual x and y coordinate values. To determine reflexive reactions, we extracted the first saccade after stimulus onset of each trial in which a stable baseline was present. Finally, only those trials with a first saccade with an amplitude of at least 0.5° of visual angle occurring between 150 and 1000 ms after stimulus onset were considered for further analyses. Non-social images and fractals were excluded since they were only used to obscure the aim of this study and to ensure active processing of all images. Of all social trials, 35.39 trials per participant (*SD* = 23.70) were excluded on average because no or only very small saccades (i.e., below an amplitude of 0.5°) were made within that timeframe. Furthermore, an average of 2.47 social trials (*SD* = 2.99) per participant had to be excluded because of missing baseline values or outliers. The average amplitude of saccades remaining in the analysis amounted to 3.10° (*SD* = 1.65°).

For each trial, we computed saccade direction and latency to evaluate to which of the four image quadrants the first saccade went and how long it took to initiate it. A saccade was considered successful if the end position of the saccade was located in the quadrant containing the social element.

### Statistical analyses

In a first step, we analyzed saccadic latencies as a function of saccade target. Therefore, we counted the number of saccades in 50 ms bins ranging from 150 ms to 1000 ms separately for saccades targeting social and non-social quadrants. These data were analyzed with a 2 (saccade target) x 17 (50 ms saccade latency bins) ANOVA on saccade frequency to investigate whether frequencies of saccadic latencies differed between successful and non-successful saccades.

Next, for each social feature position (left upper, left lower, right upper and right lower quadrant), percentage scores of successful saccades were calculated per participant. If saccades were not influenced by quadrant content but distributed randomly, one would expect a successful saccade percentage close to chance level (25%). In order to investigate whether saccades landed significantly more often in quadrants containing social elements than chance level would suggest, we subtracted 25% from the four percentage scores of each participant. We then submitted these values to a 2 x 2 repeated measures ANOVA with the factors horizontal (left versus right) plane and vertical (upper versus lower) plane of the saccade to investigate whether the distribution differed between quadrants (main and interaction effects) as well as from chance level (intercept of the ANOVA). We repeated the same procedure for saccade targets of all social images, independent of social feature location, to test whether a chance level distribution of saccades pertained when the influence of social information was not taken into consideration.

Finally, in order to investigate whether physical saliency drove saccade direction despite our initial balancing of saliency across quadrants, we also computed a generalized linear mixed model (GLMM) using the R package lme4 [37]. Mixed-effect models are a powerful and flexible tool for statistical analysis as they contain both fixed and random effects allowing the modelling of correlated and potentially non-normal data [38]. Our response variable described whether a quadrant of the presented image was looked at or not. Since this is a binary event, we chose a model with a binomial error distribution and the probit-link function. After being transformed to have a mean of 0 and a standard deviation of 1, the binary variable ‘social content of quadrant’ (social content or non-social content) and the numeric variable ‘saliency of quadrant’, together with their interaction term, were included as fixed predictors into the model. As other algorithms have proven more successful in the prediction of visual exploration patterns than the one developed by Itti and Koch [24], we decided to compute saliency scores of each quadrant using the Graph-Based Visual Saliency algorithm [39] which performed very well in the prediction of human gaze in a recent study comparing ten computational models of saliency [26]. The pattern of results remains similar when relying on the Itti and Koch algorithm [24]. The relative saliency of each quadrant was calculated by dividing the summed saliency score of each quadrant by the summed saliency score of the entire image. To account for variability between subjects and scenes, participant ID and image ID were entered as random intercepts. The size of the beta coefficients was considered to evaluate which predictor influenced saccade direction more prominently. From each trial, information of the looked-at quadrant entered the model and, additionally, one non-looked-at quadrant was chosen randomly to prevent model bias. As the random process of choosing the non-looked-at quadrant could potentially affect the significance of the results, we decided to use a bootstrapping procedure to test the validity of our model. Correspondingly, the process of randomly choosing quadrants to enter the model was repeated 2000 times and 2000 respective GLMMs were computed. We could then calculate an empirical 95% confidence interval for the beta coefficient of each predictor based on the 2000 results of this procedure. Beta coefficients were considered as significantly different from another if the confidence intervals did not overlap.

## Results

### Task performance

In order to investigate whether participants paid full attention during the experiment, we calculated task performance by dividing the number of successful responses by the number of presented test stimuli. All participants had 100% accuracy in responding to test stimuli and a low false alarm rate (i.e., behavioral responses to non-fractal images, *M* = 0.3%, *SD* = 0.8%).

### Eye movement data

**Fig 2:**
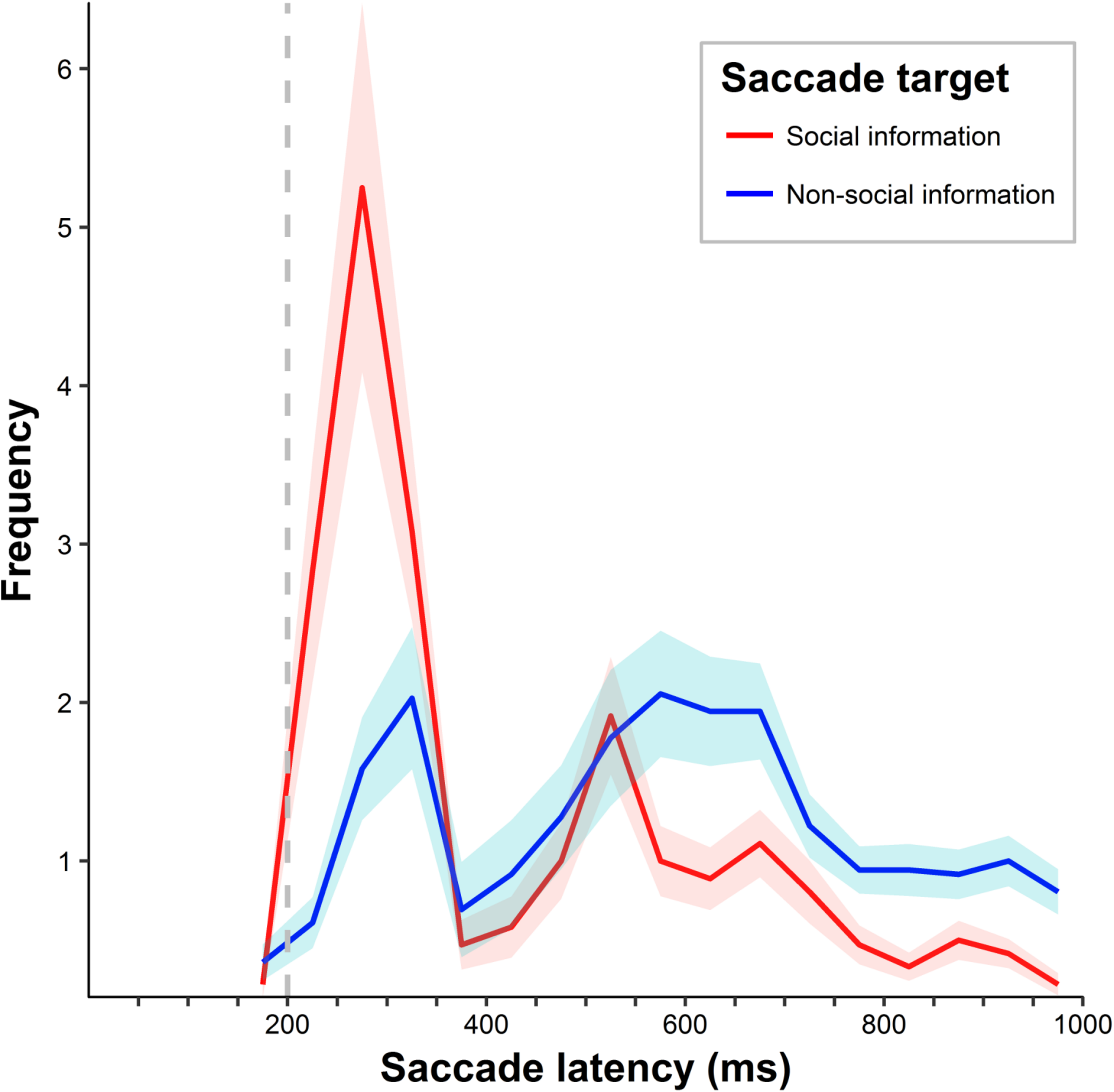
Latencies of successful and non-successful saccades. Distribution of the mean frequency of successful saccades (towards social information, in red) and non-successful saccades (towards non-social information, in blue) per 50 ms latency bin. Shaded areas are defined by the standard errors of the means. The dashed grey line indicates stimulus offset.

Overall, subjects responded swiftly to the appearing stimuli as reflected by a mean saccade latency of 467.13 ms (*SD* = 224.92 ms). A 2 (saccade target) x 17 (50 ms saccade latency bins) ANOVA on saccade frequency revealed a significant interaction between saccade target and saccade latency bin (*F*(16,560) = 11.17, *p* < .001, η_p_^2^ = .24). Fig 2 suggests a bimodal distribution of saccade latencies with saccades towards social quadrants occurring earlier as compared to saccades targeting non-social quadrants. The analysis also revealed a main effect of saccade latency bin on saccade frequency (*F*(16,560) = 9.27, *p* < .001, η_p_^2^ = .21) but no main effect of saccade target (*F*(1,35) = 0.00, *p* = .98, η_p_^2^ = .00).

**Fig 3:**
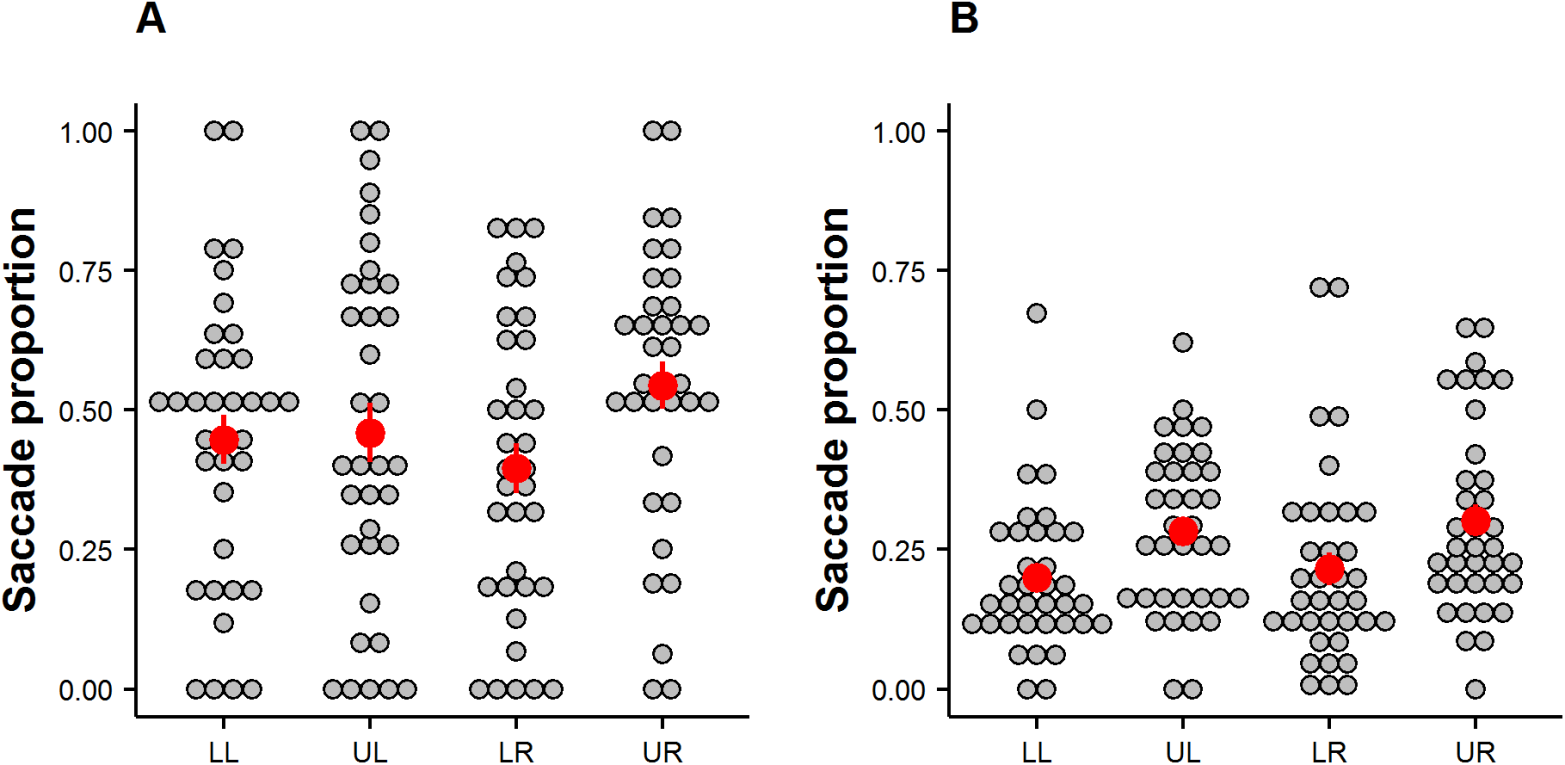
Saccade proportions. (A) Proportion of successful saccades (terminating in the quadrant in which social features were displayed). Here, proportions were calculated for all trials in which social features appeared in one of the four quadrants (LL = lower left, UL = upper left, LR = lower right, UR = upper right). Each circled dot represents one participant. Dark red dots denote the mean proportion of all participants and error bars depict the standard error of the mean. (B) Proportion of saccades terminating in one of the four quadrants (LL = lower left, UL = upper left, LR = lower right, UR = upper right) for all social scenes.

Although saccade frequency varied substantially across individuals, the 2 x 2 ANOVA, investigating the percentage scores of saccades to quadrants with social information, revealed an intercept significantly different from 0 (*F*(1,33) = 66.39, *p* < .001, η_p_^2^ = .67) indicating that the preference for quadrants containing social information was above chance level (see Fig 3A). We also observed a significant interaction effect between vertical and horizontal planes (*F*(1,33) = 4.16, *p* = .049, η_p_^2^ = .11) which suggests that a certain quadrant was preferred more than others. Indeed, Tukey post-hoc tests revealed that, when the social feature appeared on the right side of the image, participants looked significantly (*p* < .05) more often at the upper than at the lower quadrant. We did, however, not observe a main effect of horizontality (*F*(1,33) = 0.001, *p* = .97, η_p_^2^ < .001) and only a trend-level main effect of verticality (*F*(1,33) = 3.46, *p* = .07, η_p_^2^ = .09).

In contrast, a 2 x 2 ANOVA taking into account the general direction of saccades independent of social element location did not reveal an intercept significantly different from 0 (*F*(1,35) = 1, *p* = .32, *η*_*p*_^*2*^ = .03). There was, however, a significant effect of verticality (*F*(1,35) = 6.86, *p =* .01,*η*_*p*_^*2*^ = .16) as observers generally tended to look up more frequently than down (see Fig 3B). We did not observe an effect of horizontality (*F*(1,35) = 0.26, *p* = .61, *η*_*p*_^*2*^ = .007) nor an interaction effect between horizontality and verticality (*F*(1,35) = 0.01, *p* = .93, *η*_*p*_^*2*^ < .001).

In addition, we examined whether the saliency of a quadrant predicted saccade direction despite our initial balancing of stimuli. The bootstrapping procedure, which we employed to validate our general linear mixed model, revealed that both social content (mean β = 0.45) as well as saliency of a quadrant (mean β = 0.18) significantly predicted whether a quadrant was looked at (see Table 1). Since the mean of the social content beta coefficient was almost three times as large as the respective coefficient of saliency and since both confidence intervals did not overlap, these results suggest that the social content of a quadrant had a significantly greater influence on saccade direction. Importantly, there was no interaction effect between these predictors (mean β = -0.02) suggesting that the influence of these predictors was additive.

**Table 1:** Bootstrapping results.

	Mean	2.5%	97.5%
**Social Content**	0.45	0.42	0.48
**GBVS-Saliency**	0.18	0.14	0.21
**Interaction**	-0.02	-0.06	0.02

Mean and the 95% Confidence Intervals for the fixed effects ‘social content’ and ‘saliency’ and their interaction calculated on the basis of 2000 iterations.

## Discussion

In the present study, we showed that our ability to swiftly attend to humans in our surroundings seems to be reflexive. Participants briefly viewed photographs of naturalistic scenes with and without social features for 200 ms while their eye movements were being recorded. Analyses revealed that participants made significantly more first eye movements to regions containing social features than a chance level distribution of first saccades would suggest. Additionally, although saliency also drove saccade direction, the social content of an image region better predicted the target of the first saccade than its saliency, confirming that social features partially override the influence of low-level physically saliency on visual orienting. As a presentation time of 200 ms is too brief for voluntary shifts of attention to occur (as reviewed by [40]) and since such brief stimulus duration did not allow for a detailed visual exploration of the scene, our results substantiate the notion of a reflexive component of social attention.

Our main finding, that quadrants containing social features are prioritized as early as the first saccade after stimulus presentation occurs, provides novel insight into the mechanisms of social attention. Participants made significantly more first saccades to quadrants containing social information than towards other quadrants even though saliency was balanced across quadrants. Importantly, participants received no specific instructions prompting saccades but were simply told to respond with a button press when rare test stimuli (i.e., fractals) were shown. Our results consequently emphasize the reflexive nature of social attention. Admittedly, we are not the first to address the time course of social attention. However, previous studies have frequently used impoverished stimuli and experimental designs from which inferences about field conditions were more difficult to draw. Typically, social features were taken out of their context and contrasted with other isolated social features or isolated inanimate objects. In a study by Theeuwes and van der Stigchel [6], for example, participants viewed photographs of isolated faces next to photographs of appliances for 200 ms, after which an arrow, indicating the direction in which an eye movement should be made, appeared. Eye movement reactions were delayed when arrows pointed to the location where a face was previously shown. This finding was interpreted as an indicator of inhibition of return (IOR) which can be used as a diagnostic tool in visual attention to identify reflexively attended locations [41]. As IOR was greater for arrows pointing to face locations, Theeuwes and van der Stigchel concluded that attention to faces entails a reflexive component. Similarly, in a dot-probe study, isolated real faces were presented next to inanimate objects for 100, 500 or 1000 ms and participants had to promptly respond to a subsequent target appearing either at the face or at the object location [20]. Participants were quicker in detecting targets appearing at previous face locations, providing further evidence for exogenous social attention. While our results are generally in line with these findings, we were able to investigate social attention with stimuli of higher ecological validity. Simplified social stimuli neglect many aspects of a real social scene–first and foremost, the competition between different elements in a scene [9]. In our experiment, all stimuli depicted complex naturalistic scenes in which social features competed with several low-level salient non-human features. Consequently, we were able to show that social features are attended reflexively even when being surrounded by physically salient information.

Further support for the reflexive nature of social attention was provided by the mere observation of saccades in our study. Besides detecting fractals by a button press, participants did not receive any additional instructions. Hence, the observed saccades that did not allow for further stimulus exploration served no particular purpose but were reflexively triggered by the appearing stimuli. Interestingly, an investigation of saccade latencies revealed that saccades towards social information were significantly faster than saccades to image regions without social information, thus corroborating the notion of a reflexive component in social attention. These results are also in line with previous studies suggesting a dichotomy between reactive short-latency saccades and higher order saccades which display relatively longer latencies (e.g. [42,43] in natural scenes, for a general review see [44]). Accordingly, reactive saccades are believed to reflect bottom-up processes regulated by subcortical circuits, specifically the superior colliculi [45,46]. However, there is currently no consensus in the literature as to which latencies reactive saccades typically exhibit (suggestions for humans vary from 60–100 ms after image onset [43] to ~ 180 ms [47]). They are considered distinct from saccades with relatively longer latencies which, in turn, are thought to involve cortical top-down processing. Although successful saccades in our study, on average, took place slightly later than 180 ms, the difference observed between successful and non-successful saccadic latencies might be related to these different saccade types. Hence, earlier saccades which predominantly targeted social information in the current study might largely reflect reflexive, bottom-up processes, whereas later saccades mostly targeting non-social information might be further modulated by top-down, goal-driven mechanisms.

The present study revealed that social features influenced saccade direction significantly more than low-level salient features of the image. In agreement with saliency-based prediction models, saliency contributed significantly to saccade direction (mean ß = 0.18), yet social content had an even greater influence (mean ß = 0.45) thus partially overriding the influence of saliency. Importantly, no interaction between these two predictors could be observed in our model suggesting that these effects are truly additive. The studies that previously investigated social attention and saliency in complex naturalistic scenes (e.g. [11,13,15,33,34,48]) also found a prioritization of social features versus low-level salient objects in a scene. Notably, our study complements these observations in three important points: (1) our presentation time was considerably shorter ensuring that we can reliably test for a reflexive component of attention, (2) earlier studies focused on the investigation of human body parts preferences (e.g., eyes versus head) and therefore usually presented humans in the center and foreground of the image, and (3) previous studies frequently relied on older and less efficient saliency algorithms. Specifically, up until now, first fixations were frequently used as a measure of early attention [11–13,16,48]. As voluntary attention can occur as early as 300 ms after stimulus presentation (as reviewed by [40]), first fixations on specific image locations cannot be warranted as reflexive when using relatively long presentation times (≥ 2 s). We avoided this ambiguity by presenting the images for mere 200 ms which reduces the impact of higher-order processes on visual orienting. Moreover, in contrast to most previous studies [11,12,34], we carefully balanced physical saliency across social and non-social quadrants per participant prior to data collection to control for the relative saliency of social features in the currently used visual scenes. In previous studies, humans frequently took up a large and central part of the image, which can potentially explain why some failed to find any contribution of saliency to saccade direction [11]. Lastly, earlier studies frequently relied on the saliency algorithm by Itti and Koch [24] which has performed poorly in a recent review comparing different algorithms in their prediction strength [26].

One short-coming of the present study is that we used the Itti and Koch algorithm for our initial balancing of saliency across quadrants as we tried to conform to the most prominent model in the field. However, the use of the Itti and Koch algorithm was restricted to experiment preparation and for our later analysis, specifically the investigation of the contribution of saliency on eye movements, we opted for the better-performing GBVS algorithm [39]. Furthermore, it needs to be noted that while we attempted to balance saliency across social quadrants and the remaining parts of the image, this balancing was based on ranks and we can thus not guarantee that social and non-social regions do not differ slightly with regard to relative saliency density. However, we have attempted to account for a potential mismatch by investigating the contributions of saliency with a generalized linear mixed model which showed that social content contributed significantly more to saccade direction. Another drawback of the current study is the large variance of valid trials between subjects. Particularly, as we did not explicitly instruct participants to make saccades, the number of saccades per participant varied greatly. Ideally, all participants would have contributed equally to our results but our findings suggest that social information is prioritized even if some subjects react with only few saccades. Lastly, by using natural scenes, we presented ecologically valid stimuli which allowed us to shed some light on the potential mechanisms underlying gaze behavior in real-life social situations. However, we are still unable to draw strong inferences about real social interactions and it is therefore crucial that future investigations, possibly by means of mobile eye tracking or virtual reality, address gaze behavior in an interactive context to gain a more coherent understanding of social attention (see also e.g. [8]).

A great proportion of the existing literature on social attention focused on gaze following, primarily on the question whether another person’s focus of attention, indicated by their eyes being turned to one side or another, can exert a reflexive influence on visual orienting of the observer. To what extent are our findings conformant to existing gaze orienting literature? While initial studies provided evidence for a reflexive shift of attention following gaze cues (e.g. [49,50]), follow-up studies suggest that attention to gaze cues is not mediated by an exogenous mechanism (e.g. [51], also see [52]). Various studies have shown that targets are detected much faster at locations looked at by others than at non-attended locations, which, given the brief presentation time of these stimuli, led credence to the idea that attentional shifts following gaze-cues could be considered reflexive. However, evidence from a study with patient EVR who suffered from frontal lobe damage and is able to exert exogenous but not endogenous attention undermines this assumption [53]. When presented with gaze cues, EVR fails to exhibit the typical faster response to validly cued targets suggesting that gaze following is not exogenous in nature. This finding is further corroborated by a study of Itier and colleagues in which participants either had to judge gaze direction of a presented head or the direction of the head itself [51]. In the gaze task, participants directed ~90% of their first saccades to the eyes of the face, whereas only ~50% of initial saccades were directed to the eyes in the head task. If attention to gaze orienting was truly reflexive, gaze patterns should not differ between tasks.

With regard to the results of the current study, one could speculate that attention to social features is reflexive whereas attentional shifts following gaze cues might represent a secondary mechanism. Humans might reflexively attend the presented faces and employ additional resources to respond to gaze cues. Future research should therefore focus on the investigation of the neural mechanism underlying reflexive social attention and gaze orienting to clarify whether distinct neural substrates are recruited by these tasks. Finally, it needs to be noted that the validity of typical gaze cuing tasks has been recently put to question [8,54]. While gaze cues were seen to elicit similar behavioral responses as arrows in the typical simplistic laboratory designs, effects of both stimulus types were not replicated to be similar using naturalistic stimuli [12]. Instead, depictions of humans were heavily prioritized over arrows in complex scenes, which is in line with the fast selection of human features observed in our study. This discrepancy between behavioral responses to complex versus simplistic stimuli again emphasizes the necessity to render psychological experiments more ecologically valid.

To conclude, in the present study we observed that social features in complex naturalistic scenes are attended reflexively. In addition, we were able to show that social features have a significantly greater impact on first saccade direction than low-level saliency. These results therefore argue against the generalizability of saliency-based models of attention and for a crucial impact of social information on early human visual attention.
